# The Antiapoptotic RBM5/LUCA-15/H37 Gene and Its Role in Apoptosis and Human Cancer: Research Update

**DOI:** 10.1100/tsw.2006.268

**Published:** 2006-12-28

**Authors:** Mirna M Maarabouni, Gwyn T Williams

**Affiliations:** Institute for Science and Technology in Medicine, Keele University, Keele, Staffs, ST5 5BG, UK

**Keywords:** T-lymphocytes, LUCA-15, RBM5; H37, RNA-binding proteins, cell death, cell proliferation

## Abstract

The candidate tumour-suppressor gene, LUCA-15/RBM5/H37, maps to the lung cancer tumour-suppressor locus 3p21.3. The LUCA-15 gene locus encodes at least four alternatively spliced transcripts that have been shown to function as regulators of apoptosis, a fact which may have major significance in tumour regulation. This review highlights recent evidence that further implicates the LUCA-15 locus in the control of apoptosis and cell proliferation, and focuses on the observations that confirm the tumour-suppressor activity of this gene.

## INTRODUCTION

The molecular control of cancer is complex. The dysregulation of a whole range of normal cellular systemic processes contributes to the development of cancer. These processes include cell proliferation[1], cell-cell communication[2], DNA repair[3], chromosome stability[4], cell motility[5,6], apoptosis[7], and others. Despite impressive progress in recent years in understanding the molecular basis of cancer, many crucial genes remain to be identified. One or more such genes are located at the human chromosomal locus 3p21.3, which is strongly associated with lung cancer and many other cancers including head and neck, renal, breast, and female genital tract[8,9]. More than 90% of freshly microdissected primary lung cancers display loss of heterozygosity (LOH) of 3p21.3, and the fact that no single locus of this region appears to be consistently affected by LOH suggests that the region harbours more than one tumour-suppressor gene (TSG)[10,11]. The search for TSGs in this region has, therefore, been the focus of extensive investigations over the past 10 years, and many genes mapping to this region have been cloned[12,13,14].

LUCA-15 is among the 35 genes located within the 370-kb, overlapping, lung cancer, homozygous deletion region at 3p21.3[12]. Timmer et al. and Oh et al. reported the cloning of the same gene, naming it RBM5 (RNA-binding motif protein 5) and H37, respectively[8,9]. In this mini-review, the effects of LUCA-15 are discussed in the context of regulation of apoptosis, cell proliferation, and involvement in malignancy. The growing literature on this subject strongly suggests that LUCA-15 plays an important role in the regulation of cell proliferation, apoptosis, and cancer, thus elucidation of its mechanisms of action should advance our understanding both of apoptosis and of oncogenesis.

## LUCA-15: AN RNA-BINDING PROTEIN

LUCA-15 was initially cloned as a putative TSG mapping to 3p21.3[8,12] and encoded a number of alternative splice variants[23,25,26]. Full-length LUCA-15 mRNA contains an open reading frame of 815 amino acids and is translated *in vivo* into a predicted protein product of ~90 kDa[8]. It was designated RBM5 due to the presence of two RNA-binding domains (RBD), also recognised as RNA recognition motif (RRM), within its primary sequence. Other functional motifs in the LUCA-15 structure include two putative zinc-finger DNA binding motifs, two bipartite nuclear localisation signals, and a G-patch domain (a conserved domain in eukaryotic RNA-processing proteins and type D retroviral polyproteins)[15], suggesting a role for LUCA-15 in RNA processing and its localisation to the nucleus. The N-terminal fragment of human LUCA-15 containing the RNA binding domain[16] and an epitope-tagged LUCA-15 protein were found to interact preferentially with poly (G) RNA homopolymer *in vitro*[17]. In addition, the C-terminal region of LUCA-15 contains several domains including a glutamine rich domain (362-385), which is thought to serve as protein–protein interaction site in certain RNA- and DNA-binding proteins[17].

A number of proteins having one or more RRM domains have been identified (reviewed by Sutherland et al.[29]). Those include RBM6, an immediate telomeric neighbour of RBM5, also a candidate TSG, which shares 30% amino acid sequence identity, indicating that they are likely to be part of the same gene family and arose through gene duplication[7,8,16,19,20,21]. Another RBM protein that shares high homology with RBM5 (53% at the amino acid level) is RBM10. The RBM10 gene is located on the X chromosome at p11.23[39]. No function has yet been determined for RBM10, however, its high sequence homology at the amino acid level with RBM5 and RBM6 suggests that it may play an important role in apoptosis. Another protein identified with RBM domains is RBM3, which is reported to suppress polyglutamine-induced apoptosis[40]. The observation that some RBM proteins display apoptotic modulatory effects raises the possibility that their apoptotic function could be related to their function as RNA-binding proteins. Other members of the RNA-binding protein family are already reported to be involved in various aspects of RNA synthesis, alternate splicing, RNA editing, and metabolism. Important information has emerged that defines the molecular mechanisms by which RNA-binding proteins recognise specific RNA sequences and influence the processing and function of RNA molecules[27,31,32]. The importance of these interactions was also highlighted by the discovery that RNA-binding protein abnormalities are found in several human genetics disorders[33,34].

## REGULATION OF APOPTOSIS AND CELL PROLIFERATION BY LUCA-15 CDNA FAMILY

LUCA-15 is ubiquitously expressed in human tissues[16]. Its level is reported to be high in the adult thymus and low in the foetal thymus, indicating that its expression can be developmentally regulated[16]. The LUCA-15 gene encodes a number of alternative splice variants, as identified by reverse transcription-polymerase chain reactions[29,30]. Although the LUCA-15 gene has not been shown to have an increased mutation rate in cancers, a number of lines of evidence do suggest its involvement in apoptosis and in cell cycle regulation. LUCA-15 is reported to be down-regulated in RAS-transformed Rat-1 cells, in samples from breast cancer[18], in human schwannomas[38], and in ~75% of primary lung cancers specimens[36]. In addition, overexpression of LUCA-15 in human fibrosarcoma HT1080 cells suppressed cell growth[18]. Finally, LUCA-15 was one of the antigens identified by autologous antibody in patients with renal carcinoma[22].

LUCA-15's involvement in apoptosis and malignancy has been the focus of many recent studies, with all results converging on a role for LUCA-15 as a TSG. The first piece of evidence that supports a possible role for LUCA-15 in apoptosis derives from the studies performed by Sutherland and colleagues[23]. Using an episomal retrieval, functional expression, cloning system[23], a 326-bp bone marrow cDNA fragment (termed Je2; Accession No. AF107492), which strongly inhibits Fas-mediated apoptosis in Jurkat T-cells, was identified[23]. Je2 lies within an intron of the LUCA-15 gene (Accesssion No.U23946), in the opposite orientation to LUCA-15 transcription[12]. Stable expression of Je2 in another human T-cell line CEM-C7 produced marked inhibition of Fas-mediated apoptosis and conferred protection from apoptosis induced by other stimuli (Fig. 1)[24]. Since Je2 is complementary to part of the LUCA-15 locus, it is likely to suppress apoptosis in the CEM-C7 cell line through a direct antisense effect on LUCA-15 RNA. Interestingly, the LUCA-15 polypeptides present are altered significantly in CEM-C7–Je2 stable clones[35], indicating that Je2 might function to inhibit apoptosis by interfering with the processing of LUCA-15 transcripts.

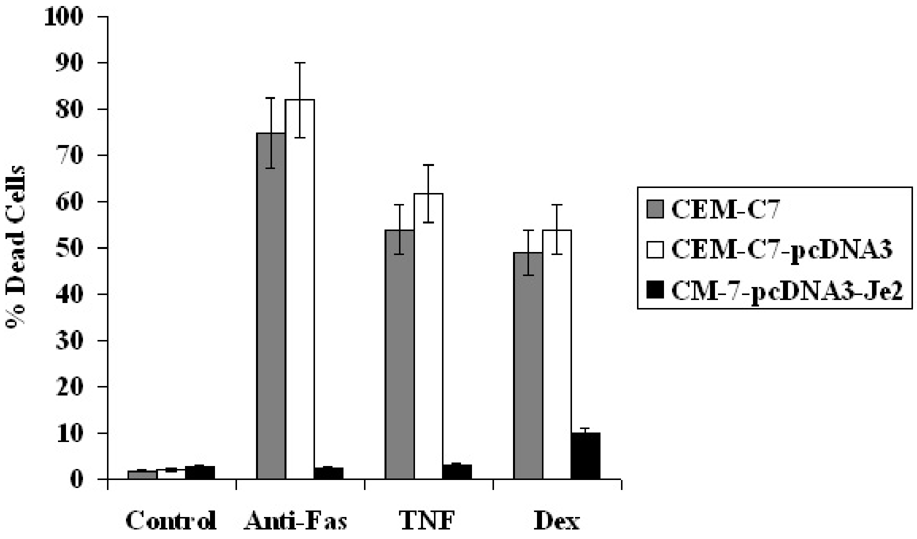

**FIGURE 1.** Je2 overexpression inhibits CD95-, TNFα-, and dexamethasone-mediated cell death. CEM-C7 cells were stably transfected with pcDNA3 vector alone or pcDNA3-Je2. Cells ($2 \times 10^5$ cells/ml) were incubated in the presence of 5 ng/ml anti-CD95, 50 ng/ml TNFα, and 10 $\mu M$ dexamethasone for 48 h. The percentage of dead cell number was determined by nigrosin dye exclusion.

Other evidence has been obtained that demonstrates that LUCA-15 is also a negative regulator of cell proliferation. Overexpression of the full-length LUCA-15/RBM5 in CEM-C7 and Jurkat T-cell lines suppressed cell proliferation both by inducing apoptosis and by inducing cell cycle arrest in $G_1$ (Fig. 2A)[25,30]. Consistently, LUCA-15 overexpression also inhibited human lung cancer cell growth (A549 nonsmall cell lung cancer line) by increasing apoptosis and inducing cell cycle arrest in $G_1$[37]. This inhibition of cell growth was reported to be associated with decreased cyclin A and phosphorylated RB, and an increase in the level of the proapototic protein Bax[37]. In addition, introducing LUCA-15 into breast cancer cells that had 3p21-22 deletions reduced both anchorage-dependent and -independent growth. LUCA-15 also is reported to suppress anchorage-dependent and -independent growth in A9 mouse fibrosarcoma cells, and to inhibit their tumour-forming activity in nude mice[36]. Most interestingly, LUCA-15 was one of the 9 genes down-regulated in metastasis and it has been included in the 17 common gene signature associated with metastasis identified in multiple solid tumour types[41]. Solid tumours carrying this gene expression signature had high rates of metastasis and poor clinical outcomes[41].

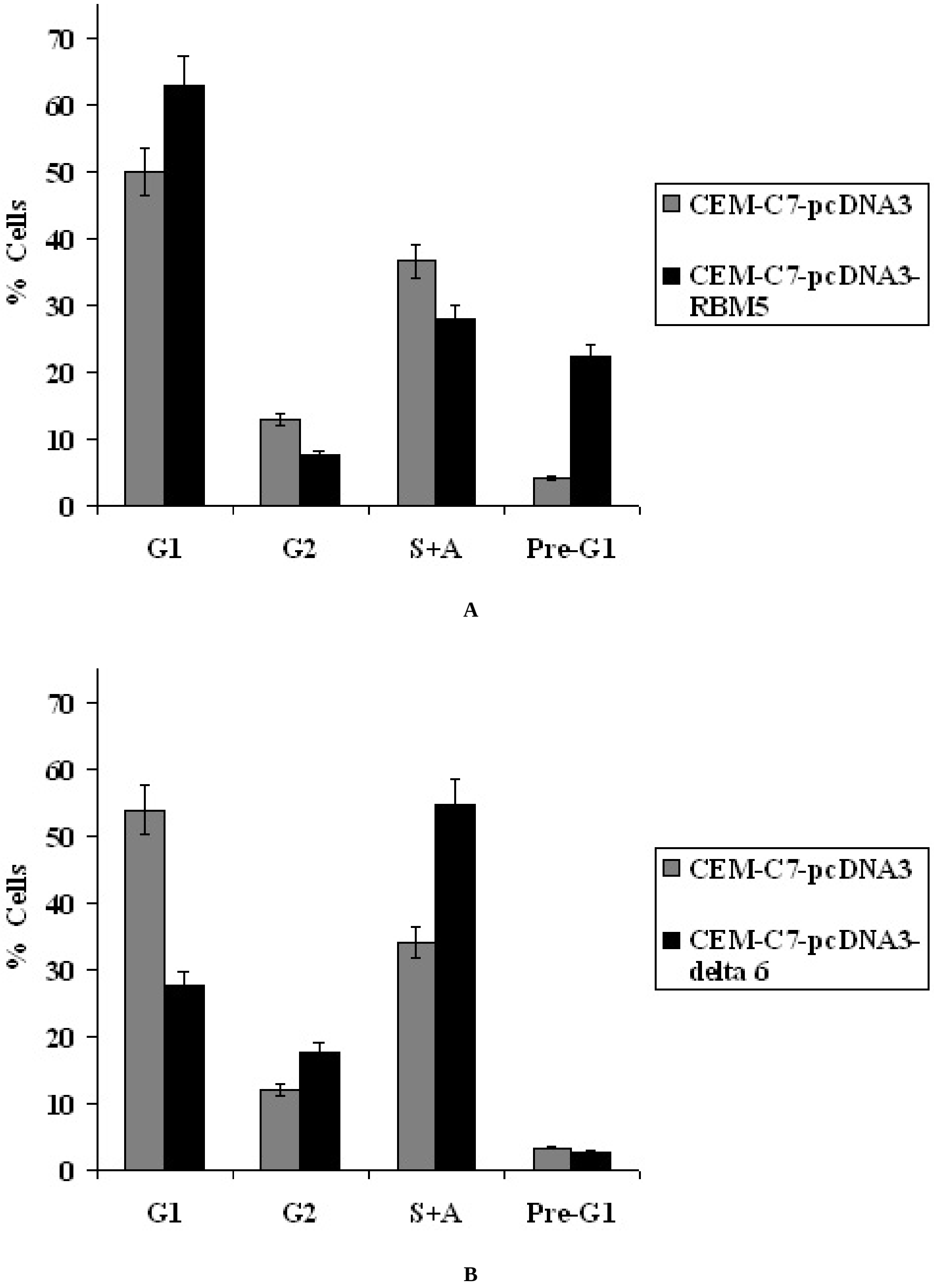

**FIGURE 2**. (A) LUCA-15/RBM5 is capable of inducing cell cycle arrest in $G_1$-M and apoptosis. DNA content from CEM-C7/pcDNA3 and CEM-C7/pcDNA3-RBM5 was quantified by propidium iodide staining of fixed cells. (B) LUCA-15Δ6 overexpression results in an increase in cells in S and G2/M phases, and a concomitant decrease in G1 cells, producing an increase in proliferation rate.

Another convincing line of reasoning derives from the fact that splice variants of the LUCA-15 gene have been shown to function as regulators of apoptosis[23]. Both alternative RNA splicing and antisense transcription occur at the LUCA-15 gene locus[23]. LUCA-15 splice variants display antagonistic properties that include promotion of apoptosis and inhibition of proliferation by transcripts encoding full-length LUCA-15 and another alternative splice variant (clone 26)[23,30]. In direct contrast, LUCA-15Δ6, a variant lacking exon 6 and producing a truncated protein, is up-regulated in some T-cell leukaemic lines and inhibits CD95-mediated apoptosis as well as accelerating proliferation (Fig. 2B)[30]. These observations suggest that changes in the level of expression of LUCA-15 or in the processing of its mRNA have profound consequences for cellular behaviour.

## EFFECTS OF LUCA-15 MODULATION ON THE EXPRESSION OF APOPTOSIS AND CELL CYCLE GENES

In order to investigate the molecular mechanisms of action of LUCA-15, microarray-based analysis of changes in gene expression caused by the modulation of the level of LUCA-15 were carried out. Among 5603 genes on cDNA microarray, 35 genes, well known for their roles in the cell cycle as well as in apoptosis, were found to be differentially expressed as a result of LUCA-15 overexpression in CEM-C7 cells[42]. Some of the LUCA-15 modulated genes include a number of cyclin-dependent kinase complexes, most notably CDK2, required during G1/S phase, and CDK inhibitor A, reported to be deleted in many cancers[42]. Three putative oncogenes are down-regulated by LUCA-15, again consistent with the role for LUCA-15 as a TSG. These are Pim-1, a serine/threonine kinase, ITPA (inosine triphosphate pyrophosphatase), and amplified in breast cancer 1 (AIB1)[42,43,44,45]. Other functionally important genes modulated by LUCA-15 are well established to play specific antiapoptotic roles, such as AIP3 (BIRC4), cIAP2 (BIRC3, MIHC), Mcl-1, a member of the Bcl-2 family of apoptosis suppressors, and TRAF1, supporting a role for LUCA-15 in apoptosis[42]. Other genes up-regulated by LUCA-15 include Stat5b, annexin I, and bone morphogenetic protein 5 (BMP5), implicated in apoptosis, immunosuppression, and organ development, respectively[42,46,47,48]. The consistent and substantial effects on such functionally important genes imply that LUCA-15 plays a critical role in the control of the cell fate.

Down-regulation of the LUCA-15 gene, achieved by the overexpression of Je2, also resulted in reciprocal changes of six key genes affected by LUCA-15, but in the reverse direction (Table 1)[42]. These genes include Stat5b, IAP homolog C, annexin I, AIB1, Pim1, and CDK2. The appearance of these complementary changes caused by LUCA-15 and Je2 provides more evidence that both these important genes are genuinely affected by LUCA-15 and that these effects are produced at physiological levels of LUCA-15.

**TABLE 1**
**Reciprocal Changes of Six Key Genes as a Result of LUCA-15 and Je2 Overexpression (Microarray-based analysis)**

| Gene Description | Cluster ID | Fold Change in RBM5 Overexpressing Clones | Fold Change in Je2 Overexpressing Clones |
|---|---|---|---|
| Stat5b | s.244613 | 4.3 (↑) | 6.7 (↓) |
| BMP-5 | Hs.1104 | 4 (↑) | 4.4 (↓) |
| cIAP2 | Hs.1277991 | 6 (↓) | 5 (↑) |
| AIB1 | Hs.225977 | 3.26 (↓) | 2 (↑) |
| Annexin I | Hs.78225 | 5.5 (↓) | 2.8 (↑) |
| CDK2 | Hs.310540 | 2.5 (↓) | 13 (↑) |

(↓) Decrease; (↑) increase.

Taken together, all of the foregoing evidence is consistent with the concept that LUCA-15 plays an important role in the regulation of cell proliferation and apoptosis, and supports a role for LUCA-15 as one of the 3p21.3 TSGs. LUCA-15 belongs to a large family of related proteins that have diverse functions in the regulation of gene expression[26]. Members of this RNA-binding protein superfamily are involved in various aspects of RNA synthesis, alternate splicing, RNA editing, and metabolism[26]. In addition, LUCA-15 contains a glutamine-rich domain, which is thought to serve as protein-protein interaction site. Thus, it is likely that LUCA-15 may regulate the activity of genes responsible for the control of the cell cycle and apoptosis by both transcriptional and post-transcriptional mechanisms. Identification of the RNA and proteins that interact with LUCA-15 should provide more information into its function.

## CONCLUSION

A number of genes, including p53, are currently accepted as *bona fide* tumour suppressors, whose mutation or deletion is associated with tumourigenesis in human cancers[28]. The ability of LUCA-15 to inhibit the growth of transformed cells fulfills one of the criteria for a tumour suppressor. In addition, LUCA-15 is located at an important site on chromosome 3, a region highly susceptible to aberrant chromosomal rearrangement and deletions, suggesting the presence of TSG at this locus[7,8,9]. The simultaneous changes in proliferation and inhibition of apoptosis brought about by dysregulation of the LUCA-15 locus are therefore likely to be of major significance in cancer development, and provide a foundation to further investigate the role of LUCA-15 in oncogenesis, to define the LUCA-15 role as a *bona fide* TSG, and to evaluate its potential as a therapeutic or diagnostic target in cancer.

## ACKNOWLEDGEMENT

This work was supported by the Wellcome Trust.
